# Purification of Active Respiratory Supercomplex from Bovine Heart Mitochondria Enables Functional Studies*

**DOI:** 10.1074/jbc.M115.680553

**Published:** 2015-12-23

**Authors:** Kyoko Shinzawa-Itoh, Harunobu Shimomura, Sachiko Yanagisawa, Satoru Shimada, Ryoko Takahashi, Marika Oosaki, Takashi Ogura, Tomitake Tsukihara

**Affiliations:** From the ‡ Department of Life Science, Graduate School of Life Science, University of Hyogo, 3-2-1, Koto, Kamighori, Akoh, Hyogo, 678-1297, Japan,; § Core Research for Evolutional Science and Technology (CREST), Japan Science and Technology Agency, Kawaguchi, Saitama 332-0012, Japan, and; ¶ Institute for Protein Research, Osaka University, 3-2 Yamadaoka, Suita, Osaka 565-0871, Japan

**Keywords:** bioenergetics, complex I, cytochrome c oxidase (complex IV), membrane protein, protein purification, Raman spectroscopy, complex III, respiratory supercomplex

## Abstract

To understand the roles of mitochondrial respiratory chain supercomplexes, methods for consistently separating and preparing supercomplexes must be established. To this end, we solubilized supercomplexes from bovine heart mitochondria with digitonin and then replaced digitonin with amphipol (A8–35), an amphiphilic polymer. Afterward, supercomplexes were separated from other complexes by sucrose density gradient centrifugation. Twenty-six grams of bovine myocardium yielded 3.2 mg of amphipol-stabilized supercomplex. The purified supercomplexes were analyzed based on their absorption spectra as well as Q_10_ (ubiquinone with ten isoprene units) and lipid assays. The supercomplex sample did not contain cytochrome *c* but did contain complexes I, III, and IV at a ratio of 1:2:1, 6 molecules of Q_10_, and 623 atoms of phosphorus. When cytochrome *c* was added, the supercomplex exhibited KCN-sensitive NADH oxidation; thus, the purified supercomplex was active. Reduced complex IV absorbs at 444 nm, so we measured the resonance Raman spectrum of the reduced amphipol-solubilized supercomplex and the mixture of amphipol-solubilized complexes I_1_, III_2_, and IV_1_ using an excitation wavelength of 441.6 nm, allowing measurement precision comparable with that obtained for complex IV alone. Use of the purified active sample provides insights into the effects of supercomplex formation.

## Introduction

Oxidative phosphorylation in mitochondria is carried out by five large multisubunit complexes: complex I^3^ (NADH dehydrogenase), complex II (succinate dehydrogenase), complex III (cytochrome *c* reductase/cytochrome *bc*_1_ complex), complex IV (cytochrome *c* oxidase), and complex V (mitochondrial F_1_F_o_ ATP synthase).

Complexes I, III, and IV catalyze the transfer of electrons from NADH to molecular oxygen and use the energy released by electron transfer to pump protons across the inner membrane. The mechanisms of electron transfer and proton pump reactions have been studied in individual respiratory complexes (1–11). However, little is known about how these complexes interact in the membrane to perform their tasks.

Two alternative models have been proposed for the arrangement of the respiratory chain complexes in the membrane. According to the random collision model (12), all components of the respiratory chain diffuse individually in the membrane, and electron transfer depends on random, transient encounters between individual protein complexes and smaller electron carriers. In the solid-state model (13) the substrate is channeled directly from one enzyme to the next within supercomplexes, which reflects a higher level of organization.

The solid-state model gained support from the discovery of supercomplexes in bovine heart and yeast mitochondria by blue native polyacrylamide gel electrophoresis (BN-PAGE) (14). The exact reasons for their presence remain elusive. It is possible that the supercomplex formation could enhance the electron flow between these complexes, stabilize individual complexes, and prevent the formation of oxygen radicals (15–23).

Single-particle electron cryomicroscopy and cryoelectron tomography has enabled three-dimensional reconstruction of supercomplexes at 19 Å, 22 Å, and 24 Å resolution using small purified samples from bovine heart (24, 25) and *Saccharomyces cerevisiae* (26). These structures have provided insight into the interactions between these complexes.

Here we report the purification from bovine heart (26 g) of amphipol-solubilized supercomplex (3.2 mg) consisting of complex I_1_, complex III_2_, complex IV_1_, and six molecules of Q_10_ (suffixes on Roman numerals indicate the number of oligomers in each complex). The purified supercomplexes exhibited KCN-sensitive NADH oxidation activities upon the addition of cytochrome *c*. Absorption spectrum and resonance Raman spectrum measurement of purified samples enabled functional investigations of the supercomplex.

## Experimental Procedures

### 

#### 

##### Preparation of Supercomplex

Bovine heart mitochondria were prepared by differential centrifugation as described in Smith (27). Mitochondria were solubilized with 6% (w/v) digitonin (Calbiochem, high purity) in 1 m sucrose, 20 mm Tris-HCl (pH 8.0) at a detergent-to-protein weight ratio of 10:1. The samples was centrifuged at 65,000 × *g* for 5 min, and the resultant supernatant was treated with amphipol (A8–35) (amphipol-to-protein ratio, 3:1) at 0 °C for 30 min. γ-Cyclodextrin (in the same detergent-to-protein ratio as digitonin) was added to the mixture, which was then incubated for 60 min at 4 °C. Precipitated material was removed by centrifugation at 65,000 × *g* for 10 min at 4 °C. The resultant supernatant was centrifuged on a stepwise sucrose gradient (1.6, 1.55, 1.5, 1.4, and 1.3 m sucrose in HEPES-Na (pH 7.8)) at 105,000 × *g* for 24 h at 4 °C. Gradients were fractionated from bottom to top, and protein content was investigated by BN-PAGE in linear gradient gels containing 3–10% polyacrylamide (Invitrogen). Protein concentration was determined by the Markwell procedure (28).

##### Sample Preparation for Spectroscopic Measurement

Protein (3.5–3.8 μm) was dissolved in HEPES-Na buffer (pH 7.8) with sucrose (0.1–1.3 m). The purified sample in oxidized form was transferred into a cylindrical quartz spinning Raman cell with a diameter of 3 mm. To reduce the protein, 10 mm of sodium dithionite was added, and the solution was agitated under anaerobic conditions.

##### Absorption Spectral Measurement

Absorption spectra from 700 to 350 nm were measured at room temperature using a spectrophotometer (U3310, HITACHI) with an attachment for the cylindrical Raman spinning cell. The sample concentration and composition were determined as follows: complex IV concentration was determined for the reduced form using an extinction coefficient of ϵ_604–630_ = 46.6 mm^−1^ cm^−1^ (29). Concentrations of *c*-type cytochrome and *b*-type cytochrome were determined on redox difference spectra using ϵ_553–544_ = 19 mm^−1^ cm^−1^ and ϵ_562–577_ = 20 mm^−1^ cm^−1^, respectively (30).

##### Resonance Raman Measurement

A 5-milliwatt He-Cd laser (IK4101R-F, Kimmon Koha) was used for Raman measurement. The laser was focused on the sample in the spinning cell (1500 rpm) from below, and Raman scattering at 90° was directed to a Raman spectrometer (Chromex, 500IS); data were collected using a liquid N_2_-cooled CCD detector (Roper Scientific, Spec-10:400B/LN). Indene and carbon tetrachloride were used as standards for frequency calibration. The resonance Raman spectra of supercomplex, mixtures of enzyme complexes, individual enzyme complexes, and sucrose solution were measured. For each sample 20 continuous 1-min measurements were carried out. After confirmation that there was no spectral change during 20 min of laser irradiation; all 20 spectra were combined into one spectrum. The contribution of sucrose to the Raman spectrum was subtracted with a reasonable coefficient. The absorption spectrum of each sample was monitored before and after Raman measurement to confirm the redox state of the sample.

##### Measurement of Ubiquinol (Q_10_) and Phospholipids

Q_10_ was extracted from 0.4 nmol of purified supercomplex samples by adding isopropyl alcohol in the presence of Q_9_ (ubiquinone with nine isoprene units) as an internal standard. Measurement of ubiquinol using LC/MS/MS (AB SCIEX QTRAP5500) was outsourced to Kaneka Techno Research. Phosphorus content of the sample solution was analyzed directly without extraction with an organic solvent, as described in Bartlett (31) and Shinzawa-Itoh *et al.* (32) with some modifications; 0.25 ml of 60% perchloric acid solution was added to 0.1 ml of sample solution containing various amounts (20–60 μg) of the protein, and the mixture was incubated overnight at 155 °C until a colorless transparent solution was obtained. After the solution was cooled to room temperature, 1.2 ml of 0.22% ammonium molybdate and 0.05 ml of 1% amidol dissolved in 20% sodium bisulfate were added, and the mixture was heated at 100 °C for 12 min. A glass ball was placed on the open end of the test tube to enable effective condensation of water vapor during the two heat treatments. Afterward, absorbance at 830 nm was determined. The phosphorus content was quantified by comparing the slope of the plot of the absorbance at 830 nm against the amount of protein with the slope of the standard sample (K_2_HPO_4_).

##### Enzyme Activity Assay

The reaction mixture (2.1–2.15 ml) contained 150 μm NADH in 100 mm potassium phosphate buffer (pH 8.0) at 20 °C in a quartz cuvette with a 1-cm light path. The cuvette was equipped with a magnetic stirrer and placed in a cuvette holder whose temperature was held constant by a circulating water system. The enzyme reaction was initiated by the addition of 10–20 μl of enzyme solution and 10 μl of 200 μm cytochrome *c* (horse heart) and followed by monitoring the decrease in absorbance at 340 nm. KCN inhibition was determined at a concentration of 2 mm inhibitor. After the addition of 4 μl of 20 mm Q_1_, piericidin A inhibition was determined at a concentration of 10 μm.

##### Preparation of Amphipol-solubilized Complex I_1_, Complex III_2_, and Complex IV_1_

Complex I was purified from bovine heart mitochondria as described previously (32, 33), except that the ammonium fractionation was omitted. The enzyme fraction, purified by anion-exchange column chromatography, was treated with amphipol (amphipol-to-protein weight ratio, 3:1). Two milliliters of 1% (W/V) β-cyclodextrin was added to the mixture and dialyzed for 60 min at 4 °C against 100 ml of 40 mm HEPES-Na (pH 7.8) containing 1% (W/V) β-cyclodextrin. The resultant mixture was centrifuged on a stepwise sucrose gradient (1.15, 1.1, 1.05, 1.1, and 0.9 m sucrose in HEPES-Na (pH 7.8)) at 105,000 × *g* for 3 h at 4 °C. Gradients were fractionated from bottom to top, and protein content was investigated by BN-PAGE in linear gradient gels containing 3–12% polyacrylamide. The enzyme activity of the amphipol-solubilized preparation, which was monitored by following NADH-Q_1_ oxidoreduction at 20 °C, was 1.5–2.1 μmol/min/nmol. This activity was 95.0–99.7% inhibited by piericidin A.

Complex III crystalline sample (24.8 mg) solubilized with 0.2% (w/v) 6-cyclohexyl-1-hexyl-maltopyranoside in 40 mm Tris-Cl buffer (pH 8.0) (34) was treated with amphipol (amphipol-to-protein weight ratio, 3:1). Two milliliters of 1% (w/v) β-cyclodextrin was added to the mixture, which was then dialyzed against 100 ml of 40 mm HEPES-Na (pH 7.8) containing 1% (w/v) β-cyclodextrin for 60 min at 4 °C. The resultant mixture was centrifuged on a stepwise sucrose gradient (1.2, 1.1, 1.0, 0.9, and 0.8 m sucrose in HEPES-Na (pH 7.8)) at 105,000 × *g* for 15 h at 4 °C. Gradients were fractionated from bottom to top, and protein content was investigated by BN-PAGE. The resultant amphipol-solubilized complex III existed as a dimer, complex III_2_, and the ratio of cytochrome *b*/cytochrome *c*_1_ was 1.95.

Crystalline complex IV (5 mg) solubilized with 0.2% (w/v) *n*-decyl-β-d-maltoside in 100 mm sodium phosphate buffer (pH 7.4) (29) was treated with 2% (w/v) *n*-octyl-β-d-glucoside at 0 °C for 120 min before the addition of amphipol (amphipol-to-protein weight ratio, 3:1). One milliliter of 1% (w/v) β-cyclodextrin was added to the mixture, which was then dialyzed against 100 ml of 40 mm HEPES-Na (pH 7.8) containing 1% (w/v) β-cyclodextrin for 60 min at 4 °C. The resultant mixture was centrifuged on a stepwise sucrose gradient (1.350, 1.338, 1.325, 1.315, 1.3, and 1.2 m sucrose in HEPES-Na (pH 7.8)) at 105,000 × *g* for 24 h at 4 °C. Gradients were fractionated from bottom to top, and protein content was investigated by BN-PAGE.

##### Immunoblotting

After electrophoresis the complexes were electroblotted onto PVDF membranes and sequentially probed with specific monoclonal antibodies against anti-cytochrome *c* (Invitrogen). Final detection was performed with secondary antibodies linked to horseradish peroxidase (GE Healthcare) using 4-chloro-1-naphthol.

## Results and Discussion

### 

#### 

##### Preparation of an Amphipol-solubilized Supercomplex from Bovine Heart Mitochondria

**FIGURE 1.**
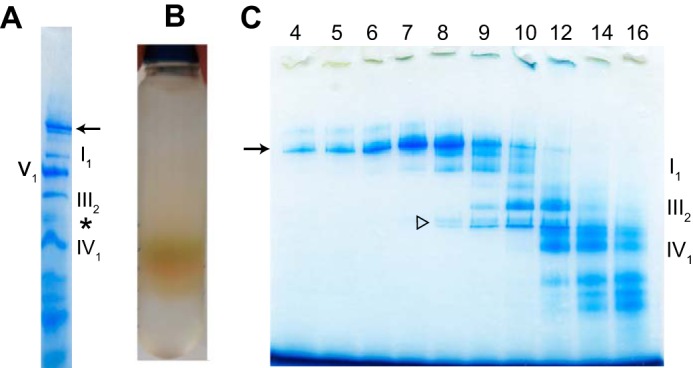
**Purification of supercomplex.** *A*, BN-PAGE pattern of bovine heart mitochondria after membrane proteins were solubilized with digitonin. *Arrow*: supercomplex. The faint broad band (*) contained a complex IV dimer. *B,* separation of supercomplex and respiratory complexes by centrifugation on a stepwise sucrose gradient. After centrifugation at 105,000 × *g* for 24 h, bands of several different colors were produced. *C*, gradients were fractionated from *bottom to top*, and protein content was investigated by BN-PAGE in linear gradient gels containing 3–12% polyacrylamide. *White arrow head*: degraded complex V during the process of sucrose density gradient centrifugation.

Fractions of bovine heart mitochondria were solubilized with digitonin and separated using BN-PAGE. This resulted in detection of a band (Fig. 1*A*) that was presumed to be a supercomplex. The supercomplex was larger than complex I, with a mass of 1000 kDa. In addition to the supercomplex and complex I, complex V_1_, complex III_2_, and complex IV monomer were also visible. The faint broad band (*) contained a complex IV dimer that was present at a markedly lower level than complex IV monomer.

Amphipol (at a weight ratio of 3:1 relative to mitochondrial protein) was added to the digitonin-solubilized fractions, γ- cyclodextrin was used to remove digitonin, and the fractions were subjected to sucrose density gradient centrifugation. After centrifugation at 105,000 × *g* for 24 h, bands of several different colors were produced, and separation of the complexes was apparent (Fig. 1*B*). Aliquots (200 μl) were taken from the bottom of the centrifuge tube, and the proteins contained in each fraction were identified by BN-PAGE (Fig. 1*C*). Protein complexes were separated by sucrose density gradient centrifugation; the bands with the darkest colors, closest to the bottom of the centrifuge tube, were determined to contain supercomplexes.

Complex V was degraded during the process of sucrose density gradient centrifugation, and bands between complex III_2_ and complex IV_1_ (about 400 kDa: *white arrow head* in Fig. 1*C*) were visible. Fractions 4–7 contained almost exclusively supercomplex. Supercomplexes solubilized by amphipol were successfully separated and purified. With a high level of reproducibility, 26 g of bovine myocardium yielded a mitochondrial fraction of 72 mg, which in turn yielded about 3.2 mg of supercomplex. Yield could be significantly increased by promptly solubilizing the prepared mitochondrial membranes with digitonin.

##### Characterization of the Purified Supercomplex

**FIGURE 2.**
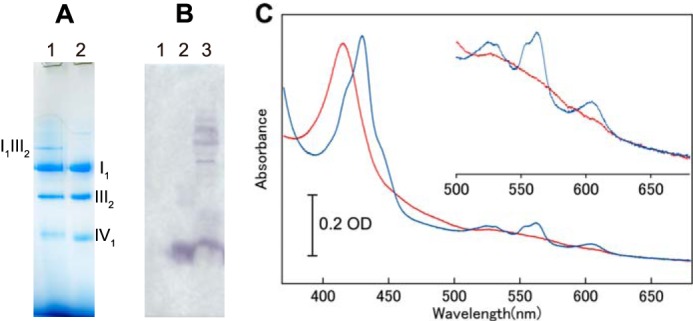
**Characterization of the purified supercomplex.** *A*, BN-PAGE pattern of treated amphipol-solubilized supercomplex (*lane 1*) and mixture of purified complexes I_1_, III_2_, and IV_1_ (*lane 2*). 11 μg of amphipol-solubilized supercomplex was treated with 40 mm HEPES-Na (pH 7.8) containing 50 mm KCl and 0.25% (W/V) lauryl maltose neopentyl glycol. The mixture contained 5.0 μg of complexes I_1_, 2.5 μg of complex III_2_ and 1.0 μg of complex IV_1_. *B*, Western blotting pattern using anti-cytochrome *c* antibody. 94 μg of supercomplex (*lane 1*), 1.0 μg of horse heart cytochrome *c*, and 120 μg of SDS-solubilized mitochondria membrane (*lane 3*) were loaded and transferred to PVDF membrane. *C*, the absorption spectra of both the oxidized complex as isolated (*red*) and the dithionite-reduced complex (*blue*).

BN-PAGE revealed a band (*arrow* in Fig. 1, *A* and *C*) that was presumed to represent a supercomplex larger than complex I with a mass of 1000 kDa. To demonstrate that this supercomplex band contained complexes I, III, and IV, amphipol-solubilized supercomplex was treated with 40 mm HEPES-Na (pH 7.8) containing 50 mm KCl and 0.25% (w/v) lauryl maltose neopentyl glycol overnight at 4 °C. After this treatment bands derived from complexes I_1_, III_2_, and IV_1_ were observed in BN-PAGE (Fig. 2*A*). The supercomplex was dissociated into complexes I_1_, III_2_, and IV_1_. The faint band, larger than complex I, contained a complex of I_1_ and III_2_; complex IV was removed from supercomplex. The presence of complex III and IV was also clearly demonstrated by the absorption spectra, whereas complex I could not be detected. However, complex I was clearly detected in BN-PAGE pattern. Together, these results show that the supercomplex consisted of complexes I, III, and IV.

Absorption spectra for the oxidized and sodium dithionate-reduced forms of amphipol-solubilized supercomplex are shown in Fig. 2*C*. The absorption spectra of the reduced form exhibited peaks at 553, 562, and 604 nm due to the presence of *c*-type, *b*-type, and *a*-type cytochrome, respectively. For this study we prepared several samples of supercomplex, with the concentration of complex IV_1_ ranging from 2.0 to 3.8 μm. We measured the absorption spectra of these samples and determined the concentrations of the cytochromes in each. The ratio of *b*-type to *c*-type cytochrome was close to 2 (1.77 ± 0.092; *n* = 8). The ratio of *b*-type cytochrome to complex IV monomer was 3.88 ± 0.35 (*n* = 8), and the ratio of *c*-type cytochrome to complex IV monomer was 2.20 ± 0.14 (*n* = 8). These findings revealed that the samples did not contain cytochrome *c* (Table 1), which was confirmed immunochemically (Fig. 2*B*). Previous research revealed a structure with one molecule of cytochrome *c* bound to the complex III dimer, and that study suggested that cytochrome *c* was present in the supercomplex (24). However, the measurements reported here strongly indicate that cytochrome *c* was not present in our supercomplex sample, possibly because the binding of cytochrome *c* and complex III was not strong enough to withstand the process of sucrose density gradient centrifugation. Complex I has a mass of 1,000 kDa, and its mobility in BN-PAGE indicated that it is unlikely to form a dimer. Thus, the prepared supercomplex consisted of complex I, complex III, and complex IV at a ratio of 1:2:1 but did not contain cytochrome *c*.

**TABLE 1:** **Concentration of cytochromes in each supercomplex samples**

Sample	*b-*Type	*c-*Type	*b-*Type/*c-*Type	IV_1_	*b-*Type/IV_1_	*c-*Type/IV_1_
	μ*m*	μ*m*		μ*m*		
1	9.46	5.15	1.84	2.23	4.24	2.31
2	5.09	2.83	1.80	1.18	4.32	2.40
3	9.16	5.08	1.80	2.68	3.41	1.90
4	9.25	4.80	1.93	2.13	4.34	2.25
5	8.20	4.67	1.76	2.14	3.83	2.18
6	8.60	5.00	1.72	2.40	3.58	2.08
7	13.70	8.57	1.60	3.89	3.52	2.20
8	13.50	7.93	1.70	3.53	3.82	2.25
Average (S.D.)			1.77 (0.09)		3.88 (0.35)	2.20 (0.14)

We then determined the amount of Q_10_ per complex IV_1_, *i.e.* the amount of Q_10_ contained in one molecule of the supercomplex. The results of this analysis revealed that the supercomplex contained 6.23 ± 1.11 (*n* = 6) molecules of Q_10_. This Q_10_ was detected only in the oxidized form but not in the reduced form. Similarly, we determined the amount of Q_10_ in the mitochondrial membrane and found that there were 6.30 ± 0.37 (*n* = 7) molecules of Q_10_ per complex IV_1_. There was no difference between the contents of Q_10_ per complex IV_1_ in supercomplex and mitochondrial membrane. These results indicate that there was no specific uptake of Q_10_ by the supercomplex and support the idea that Q exists as a common pool in mitochondria that is exchanged freely between complexes (35). The purified sample of complex I_1_ contained one molecule of Q_10_ (32). The crystalline sample of complex III_2_ prepared by our method (34) contained 1.00 ± 0.08 (*n* = 4) molecules of Q_10_ per complex III dimer. Presumably, these Q_10_ molecules bind strongly to enzymes and play a role in the function of each complex. The supercomplex purified in this study contained six molecules of Q_10_; four of these molecules facilitate electron transfer between complexes I and III, as is discussed later.

In the supercomplex individual complexes are thought to assemble via lipids, and the association of individual complexes strongly depends on the amount and composition of lipids (26, 36, 37). To estimate the phospholipid content in the supercomplex, we assayed phosphorus. The results revealed that each mol of supercomplex contained 623 ± 102 mol (*n* = 7) phosphorus. Purified complex I_1_ and the crystalline samples of complex III_2_ and complex IV_1_ contained 71, 108, and 13 mol of phosphorus, respectively (32, 34, 36). These phospholipids found in complexes I_1_, III_2_, and IV_1_ are specifically bound to the protein moiety; in other words, all of these phospholipids are intrinsic constituents of these complexes. The cryo-EM maps of the yeast and bovine supercomplexes showed that complexes I_1_, III_2_, and IV_1_ are some distance apart within the supercomplexes (24–26). In our supercomplex samples, each mol of supercomplex contained 623 mol of phosphorus, greater than the total phosphorus contents of individual complexes. The space between the individual complexes is most likely to be filled with these lipids.

Next we examined the composition of phospholipids in the supercomplex. To this end we extracted lipids from the supercomplex and qualitatively analyzed them by mass spectroscopy. As described in a previous report (36), we analyzed the lipids of each purified respiratory complex (I, III, IV, and V). This analysis revealed the presence of cardiolipin (C = 18:2), phosphatidylethanolamine (C = 18:0,20:4), phosphatidylcholine (C = 16:0,18:2) (C = 16:0,18:1), choline plasmalogen (C = 16:0,18:2) (C = 16:0,18:1), and phosphatidylglycerol (C = 16:0,18:1). These respiratory complexes contain these seven species of phospholipids in varying ratios. The types of lipids that the supercomplex contained coincided with the lipids contained in individual complexes, as previous studies have reported (36).

Several studies have reported that cardiolipin is essential for the formation of supercomplexes (37, 38). Therefore, we examined the cardiolipin and phosphatidylethanolamine content in the supercomplex: 50–90 mol of cardiolipin and 180–250 mol of phosphatidylethanolamine were present per mol of supercomplex (determined in three independent experiments). The proportions of phosphorus in these lipids relative to total phosphorus in supercomplex samples differed little from their proportions in the mitochondrial membrane; thus, it is unlikely that cardiolipin and phosphatidylethanolamine are specifically involved in these interactions. It is not clear how cardiolipin can play an important role in the interaction between individual complexes but not specifically in supercomplex formation.

##### Activity of the Purified Supercomplex

Next, we measured the oxidation of NADH by supercomplexes at 20 °C and compared the electron transfer reactions with those of a mixture of purified complexes I_1_, III_2_, and IV_1_.

**FIGURE 3.**
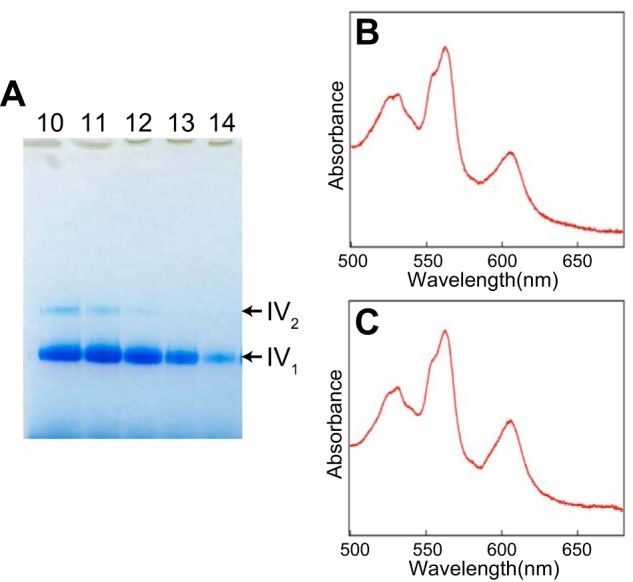
**Preparation of amphipol-solubilized mixture of complexes I_1_, III_2_, and IV_1_.** *A*, preparation of complex IV monomer. After centrifugation on a stepwise sucrose gradient, gradients were fractionated from *bottom to top*, protein content was investigated by BN-PAGE on linear gradient gels containing 3–12% polyacrylamide. Fractions 13 and 14 were collected as complex IV_1._ *B*, absorption spectra of the dithionite-reduced supercomplex. *C*, absorption spectra of dithionite-reduced mixture of complexes I_1_, III_2_, and IV_1_.

Complex IV in the supercomplex exists as a monomer. Because the enzyme purified with *n*-decyl-β-d-maltoside is present as a balance of monomer and dimer, we prepared monomer complex IV from *n*-decyl-β-d-maltoside-solubilized enzyme by replacing the detergent with amphipol and separating the sample by sucrose density gradient centrifugation, as confirmed by BN-PAGE (Fig. 3*A*). On the other hand, the mixture of each individual component of the supercomplex requires complex I_1_ and complex III_2_ in addition to the complex IV monomer. Previously, we successfully generated two- or three-dimensional crystals of complex I, complex III, and complex IV (10, 33, 34). From these highly pure samples, complex I_1_ and complex III_2_ as well as complex IV_1_ were prepared with amphipol in the same manner as in the supercomplex preparation. These enzyme complexes exhibited absorption spectra very similar to those of enzyme complexes solubilized with a surfactant. The mixture of amphipol-solubilized complex I_1,_ III_2_, and IV_1_ exhibited a spectrum almost identical with that of the amphipol-solubilized supercomplex (Fig. 3, *B* and *C*).

**FIGURE 4.**
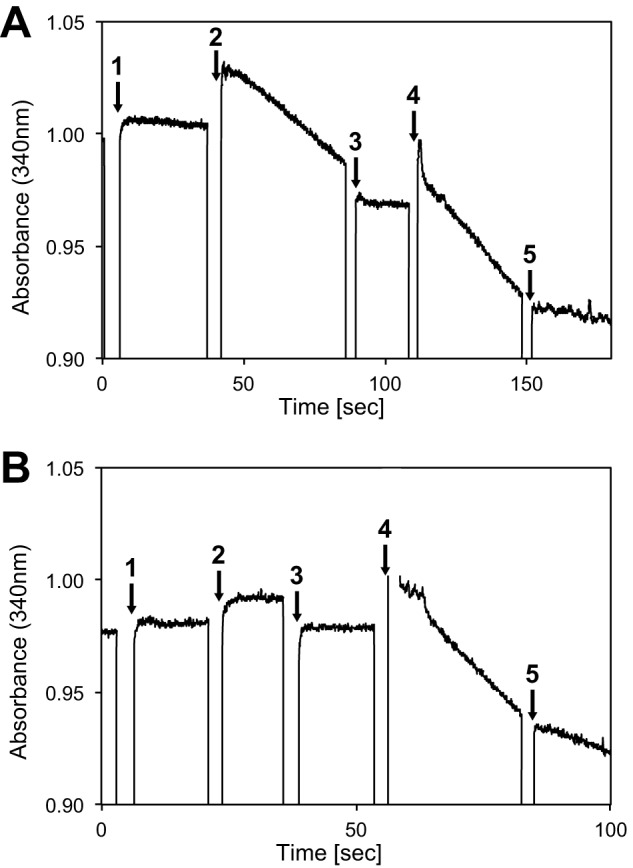
**Activity measurements of the electron transfer reaction from NADH to O_2_ of purified supercomplex (*A*) and the mixture of purified complexes I**_1_, III**_2_, and IV_1_ (*B*).** The reaction mixture contained 150 μm NADH in 100 mm potassium phosphate buffer (pH 8.0) at 20 °C. The enzyme reaction was initiated by the addition of 10–20 μl of the enzyme solution (*1*) and 10 μl of 200 μm cytochrome *c* (*2*) and followed by monitoring the decrease in absorbance at 340 nm. KCN inhibition (*3*) was determined at a concentration of 2 mm inhibitor. After the addition of 4 μl of 25 mm Q_1_ (*4*), piericidin A inhibition (*5*) was determined at a concentration of 10 μm.

In the case of the supercomplex, oxidation of NADH was not detected even when enzyme was added to 100 mm potassium phosphate buffer (pH 7.8) containing 150 μm NADH. However, when oxidized cytochrome *c* was added at a final concentration of 1.0 μm, oxidation of NADH was observed. The addition of 2 mm KCN, a complex IV inhibitor, inhibited supercomplex activity, and added cytochrome *c* was completely reduced. After the addition of 50 μm Q_1_, oxidation of NADH re-started. The addition of 10 μm piericidin A, an inhibitor of complex I, inhibited NADH oxidation by complex I in the supercomplex (Fig. 4*A*). In the case of the mixture of purified complex I_1,_ III_2_, and IV_1,_ oxidation of NADH was not observed after the addition of 1.0 μm oxidized cytochrome *c*. After the addition of Q_1_, oxidation of NADH started. The addition of piericidin A inhibited NADH oxidation by complex I (Fig. 4*B*). These findings indicate that electron transfer from NADH to O_2_ by the six molecules of Q_10_ contained in the supercomplex and the added cytochrome *c* was facilitated by complex I_1_, complex III_2_, and complex IV_1_ contained within the supercomplex. The NADH oxidation rate of the supercomplex after the addition of cytochrome *c* was 0.48–0.74 μmol/min/nmol supercomplex (determined in 4 independent preparations). Furthermore, the rate of electron transfer from NADH to Q_1_ by complex I in supercomplex, after inhibition by KCN, was 0.70–1.12 μmol/min/nmol. The rate of electron transfer from NADH to Q_1_ in mixtures of individual complexes was 0.68–1.11 μmol/min/nmol complex I in supercomplex (determined in three independent preparations). The measured level of activity was appropriate given that the six molecules of Q_10_ contained in each supercomplex transfer electrons between complexes I and III. Thus, the prepared supercomplex was active and retained this activity for at least 7 days on ice.

##### Raman Spectra of the Supercomplex and Mixture of Complex I_1_, Complex III_2_, and Complex IV_1_

**FIGURE 5.**
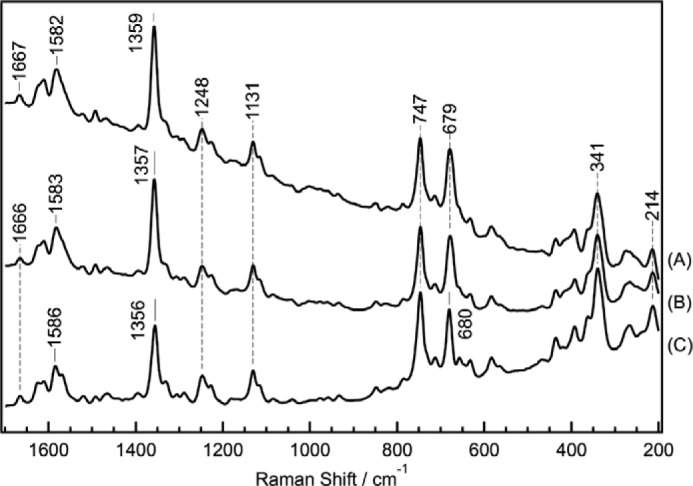
**Resonance Raman spectra of fully reduced amphipol-solubilized samples.** Supercomplex (*A*), mixture of amphipol-solubilized complex I_1_, complex III_2_, and complex IV_1_ (*B*), and amphipol-solubilized complex IV monomer (*C*).

**FIGURE 6.**
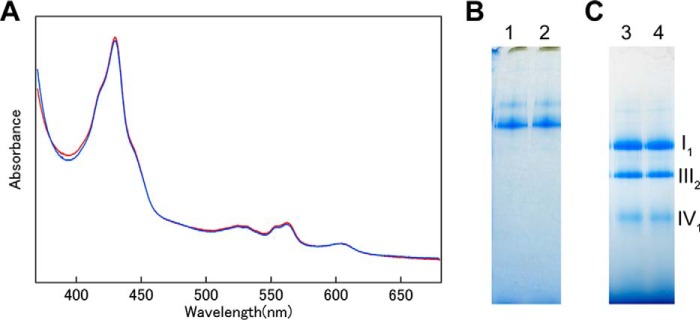
**Absorption spectra and BN-PAGE pattern before and after measurements of Resonance Raman spectra.** *A*, absorption spectra of fully reduced amphipol-solubilized supercomplex before (*red*) and after (*blue*) measurements of Resonance Raman spectra. *B*, BN-PAGE pattern of the supercomplex and *C*, mixture of complexes I_1_, III_2_, and IV_1._ *Lanes 1* and *3*, before measurement of Resonance Raman spectra. *Lanes 2* and *4*, after measurement of Resonance Raman spectra.

To determine whether the prepared supercomplex was suitable for Raman spectroscopy, with the goal of examining the effect of the intermolecular interaction of individual complexes in the supercomplex on the enzyme reaction center, we measured the resonance Raman spectra of the prepared supercomplex and mixture of prepared individual complexes. The largest body of Raman spectral data has been assembled for complex IV. Reduced heme *a* has an absorption maximum at 444 nm, so the reduced form of the supercomplex was excited using a 441.6-nm laser to measure its resonance Raman spectrum. Fig. 5 shows resonance Raman spectra of the supercomplex (A), the mixture of individual complex (B), and the monomer of complex IV (C). The resonance Raman spectrum of the monomer complex IV (Fig. 5*C*) shows the formyl ν_CH = O_ of heme *a*_3_ at 1666 cm^−1^, ν_2_ at 1586 cm^−1^, ν_4_ at 1356 cm^−1^, ν_16_ at 747 cm^−1^, ν_7_ at 680 cm^−1^, ν_8_ at 341 cm^−1^, and ν_Fe-His_ at 214 cm^−1^(39), indicating that complex IV was in the fully reduced form. Furthermore, the spectrum is identical to that of *n*-decyl-β-d-maltoside-solubilized complex IV. There was no change in the absorption spectra (Fig. 6*A*) or BN-PAGE pattern (Fig. 6, *B* and *C*) as a result of the Raman measurement, indicating that laser illumination did not induce any change in the supercomplex. The resonance Raman spectra of the supercomplex (Fig. 5*A*) and mixture of the individual complexes (Fig. 5*B*) were essentially identical to that of the monomer of complex IV (Fig. 5*C*) because the excitation wavelength was adjusted for reduced complex IV. Thus, the spectra obtained for the supercomplex and mixtures were presumed to be due to the presence of complex IV_1_ in each solution. On the other hand, the resonance Raman spectrum of the supercomplex (Fig. 5*A*) was more similar to that of the mixture (Fig. 5*B*) than that of the monomer (Fig. 5*C*). This is reasonable because the mixture contains the same amount of enzyme complexes as the supercomplex, including *b*- and *c*-type hemes, as confirmed by their comparable absorption spectra (Fig. 3, *B* and *C*). The qualities of the Raman spectra among these three samples were equivalent, and even single vibrational modes, such as ν_CH = O_ of heme *a*_3_ and ν_Fe-His_ of complex IV, were detectable in the Raman spectrum of the supercomplex, which had a mass of 1700 kDa. Thus, we can confidently say that our supercomplex and prepared individual complexes were suitable for Raman measurement. It should be possible to perform further studies to confirm the effect of supercomplex formation on each complex, *e.g.* on the dynamics of electron transfer or CO binding to the supercomplex, as a proof of O_2_ binding.

Thus far, the pseudo-atomic resolution structure of the supercomplex has been described by combining electron microscopy (cryo-EM maps) at a resolution of 19 Å and the x-ray structures of its components (24). In the supercomplex structure, individual complexes are adjacent, but there is no direct interaction between the proteins. The steric structure of the supercomplex must be determined to establish whether protein interactions are mediated by lipids.

## Author Contributions

T. T. and K. S.-I. designed the research; H. S., S. Y., S. S., R. T., M. O., T. O., T. T., and K. S.-I. performed the research; purification and biochemical studies were performed by H. S., S. S., R. T., M. O., and K. S.-I; spectroscopic measurement was performed by H. S., S. S., S. Y., and K. S.-I. S. Y., and T. T; K. S.-I. wrote the paper.

## Footnotes

3 The abbreviations used are:

complex I: NADH dehydrogenase
complex II: succinate dehydrogenase
complex III: cytochrome *c* reductase/cytochrome *bc*_1_ complex
complex IV: cytochrome *c* oxidase
complex V: mitochondrial F_1_F_o_ ATP synthase
complex I_1_: complex III_2_, complex IV_1_, and complex V_1_ suffixes of Roman numerals indicate the number of oligomers in each complex
Q_10_: ubiquinone with 10 isoprene units
Q_1_: ubiquinone with one isoprene unit
BN-PAGE: blue native-PAGE.
